# Polyurethane/silk fibroin-based electrospun membranes for wound healing and skin substitute applications

**DOI:** 10.3762/bjnano.16.46

**Published:** 2025-04-24

**Authors:** Iqra Zainab, Zohra Naseem, Syeda Rubab Batool, Muhammad Waqas, Ahsan Nazir, Muhammad Anwaar Nazeer

**Affiliations:** 1 Biomaterials and Tissue Engineering Research (BIOMATTER) Laboratory, National Textile University, Faisalabad 37610, Pakistan; 2 School of Engineering and Technology, National Textile University, Faisalabad 37610, Pakistan

**Keywords:** electrospinning, nanofibers, polyurethane, silk fibroin, skin regeneration, wound healing

## Abstract

The importance of electrospun membranes for biomedical applications has increased, especially when it comes to skin regeneration and wound healing. This review presents the production and applications of electrospun membranes based on polyurethane (PU) and silk fibroin (SF) and highlights their benefits as a skin substitute. This review also highlights the electrospinning technique used to prepare nanofibers for these biomedical applications. Silk, well-known for its excellent biocompatibility, biodegradability, structural properties, and low immunogenic response, is extensively investigated by addressing its molecular structure, composition, and medical uses. PU is a candidate for potential biomedical applications because of its strength, flexibility, biocompatibility, cell-adhesive properties, and high resistance to biodegradation. PU combined with silk offers a number of enhanced properties. The study offers a comprehensive overview of the advanced developments and applications of PU/SF composites, highlighting their significant potential in wound healing. These composite membranes present promising advancements in wound healing and skin regeneration by combining the unique properties of silk and PU, opening up the possibilities for innovative treatments.

## Introduction

The biomedical field is a revolutionary sector in healthcare and research, focused on improving human health through innovative technologies [1]. It is a broad interdisciplinary field that includes biology, medicine, engineering, and materials science, which all work together to address the intricate medical needs of societies [2]. The ultimate goal of biomedical research is to understand the human anatomy and to engineer devices that improve the patients’ life [3]. In the ever-evolving biomedical field, researchers have experimented with a wide range of materials to address various conditions including trauma and aging [4]. Both natural and synthetic materials are important for the development of implants, medical devices, and drug delivery systems that solve challenging issues from health to medicine [5]. Because of innovations in tissue engineering, genetic engineering, and nanotechnology, scientists can now see into the innermost functions of living organisms with a level of clarity never before possible [6–7]. In nanotechnology, different nanofiber production techniques are used such as template synthesis, electrospinning, solution blowing, drawing, thermally induced phase segregation, and self-assembly [8–9]. Among these techniques, electrospinning is the most versatile and provides the best control over fiber morphology and structure [10]. Electrospinning is a straightforward and adaptable technique that can be used to directly spin polymeric solutions into nanofibers that are at least 100 times thinner than fibers created using more conventional techniques [11].

Nanofibers are considered promising materials because of their size and structure, making them suitable for drug delivery, tissue engineering, tissue scaffolding, and other biomedical applications [12]. They exhibit distinct chemical and physical properties that distinguish them from macroscale structures. The properties of nanofibers such as their high specific surface area, large surface-to-volume ratio, large length-to-diameter ratio, porous membrane structure, and their ability to mimic the extra-cellular matrix (ECM) of natural tissues make them a suitable material for wound dressing and skin substitute applications [13]. Various materials including natural polymers, synthetic polymers, or composites have been used to create electrospun membranes. This review focuses on silk fibroin/polyurethan-based electrospun membranes. Silk fibroin (SF) is a naturally occurring biomaterial made of proteins, produced by silkworms, beetles, mites, and spiders [14]. Silk is an FDA-approved biomaterial for medical applications [15]. SF has distinctive physical, chemical, and mechanical properties [16]. Its characteristics, including biocompatibility, biodegradability, elasticity, solubility in water, and ease of processing into nanofibers, make it an ideal material for biomedical applications including the reconstruction of bone and cartilage [17–18]. Polyurethan (PU) is a very flexible, long-lasting, and reliable material. It is versatile and can be used in almost every field of work. PU can also be used in biomedical applications because of its biocompatibility [19]. It can be used for developing scaffolds for tissue engineering that work efficiently [20]. Electrospun membranes of silk/PU composites have remarkable qualities that make them suitable for wound healing [21]. Their porous shape allows for nutrient transport and gas exchange, which supports tissue regeneration and cell proliferation [22]. Furthermore, they have mechanical qualities that are similar to those of natural tissues, promoting and protecting the healing process [23].

## Review

### Wound healing and skin regeneration

**Figure 1:**
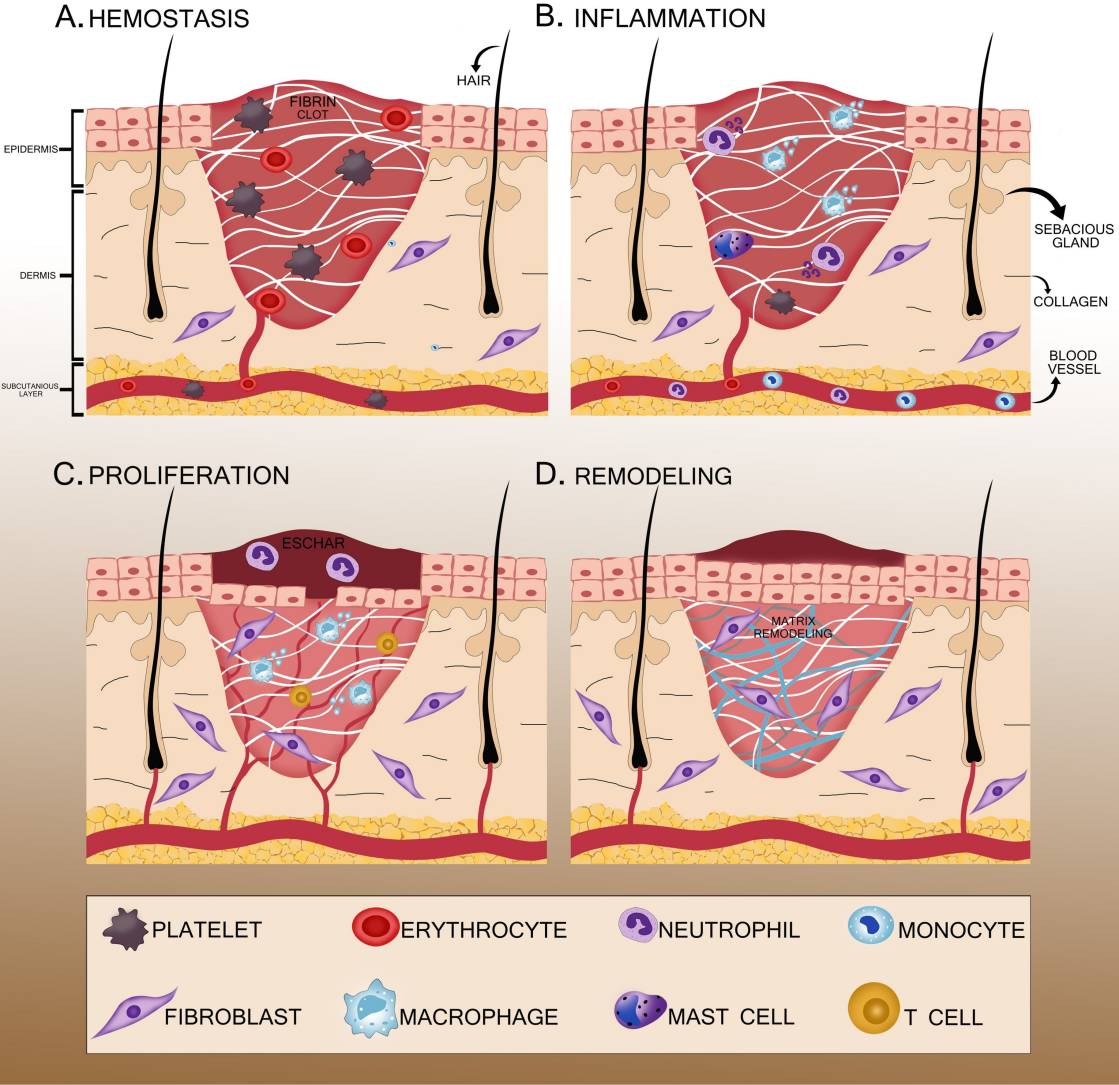
Stages of wound healing. (A) Hemostasis: Blood vessels constrict and clots stop the bleeding. (B) Inflammation: The focus is on preventing infection. (C) Proliferation: Fibroblasts form granulation tissue, angiogenesis produces new blood vessels, and keratinocytes close the wound. (D) Remodeling: Fibroblasts reorganize the extracellular matrix, blood vessels retract, and myofibroblasts contract the wound.

The skin is a vital, protective, and regulatory organ. It protects us from microbes and environmental harmful elements, controls body temperature, and gives the sensation of heat, cold, pain, pressure, and touch [24]. Epidermis, dermis, and hypodermis are the three primary layers of the skin [25]. The integrity of the skin can be compromised by trauma, congenital anomalies, burns, or because of a chronic defect. After a skin injury, it is imperative to restore skin integrity for homeostasis function and protection from microbes [26]. Wounds can be acute or chronic. Acute wounds heal in a matter of weeks, while chronic wounds take several months to heal [27]. As soon as wounds occur, the body starts the healing process by rushing blood to the affected area, which helps to remove dirt and bacteria [28]. Wound healing refers to the complete process of tissue restoration, whereas skin regeneration focuses on the replacement of skin cells [29]. Multiple cell types including immune cells, fibroblasts, and keratinocytes work together to repair wounds [30]. Skin regeneration occurs mainly through the proliferation and differentiation of keratinocytes, the major cell type of the epidermis, that move to cover the wound and generate new skin tissue [31]. After skin injury, a series of processes known as the “wound healing process” occurs to regain the skin’s structural and functional properties. As shown in Figure 1, the wound healing process consists of four stages, namely, hemostasis, inflammation, new tissue generation, and remodeling of tissues or maturation [32].

#### Hemostasis

After an injury, the first response is contraction blood vessels and coagulation of blood to reduce blood and fluid loss. Platelets play a key role in hemostasis function. Platelet receptors interact with ECM proteins, such as collagen, fibronectin, and von Willebrand factor, and promote adherence to the walls of blood vessels [33]. Thrombin activates the platelets and releases bioactive molecules that support coagulation [34]. An insoluble clot is formed by fibronectin, fibrin, vitronectin to prevent bleeding. This eschar also serves as shielding against microbes and as scaffold for immune cells. Also, it stores growth factors and cytokines to promote healing [35]. Platelets bring the immune cells to the injury site either through direct capturing or by the release of the secretome. The secretome activates keratinocytes and fibroblasts of the resident skin cells [36].

#### Inflammation

At this stage of wound healing, skin regeneration begins and continues till the end. Skin regeneration is essential for maintaining homeostasis, avoiding infection, and repairing the skin’s barrier function [37]. The inflammation process kills pathogens, prevents the wound from infection, and clears the debris [38]. Necrotic cells and injured tissue release signals that trigger the immune response. The resident immune cells such as Langerhans cells, mast cells, T cells, and macrophages respond to the injury-induced signals. Pro-inflammatory molecules, chemokines, and cytokines regulate the leucocytes at the injury site. Neutrophils (leucocytes) remove pathogens and necrotic tissues by phagocytosis, antimicrobial peptides, and the release of active oxygen species, proteolytic enzymes, as well as eicosanoids [39]. Excessive and uncontrolled inflammation causes delayed healing. Insufficient immune cell recruitment can also hinder the healing process. To promote fast healing, immune cell response must be appropriate, beginning with increased recruitment of cells and followed by effective clearance of the wound site. Once the infection is addressed, neutrophils are cleared by macrophage efferocytosis, apoptosis, or return to blood vessels [40].

#### Proliferation

Granulation tissue development, re-epithelialization, and neovascularization are features of the proliferative phase. This period may last several weeks [41]. Fibroblasts, keratinocytes, macrophages, and endothelial cells are heavily activated during the proliferation stage, which helps in matrix deposition, wound closure, and angiogenesis. In wound healing, the formation of granulation tissue occurs, which is composed of cellular and fibril matrix [42]. Fibroblasts synthesize the fibrillar components, myofibroblasts help in wound contraction, and endothelial cells are involved in neo-angiogenesis. The re-epithelization process involves proliferation and migration towards the wound area and fills the wound with granulation tissues. In this matrix, keratinocytes live, move, and proliferate to close the wound [43].

#### Remodeling phase

Remodeling is the last phase of wound healing, which ends with the formation of scars and clearance of immune cells from the epidermis. All processes from the previous stages are completed in this phase [44]. When a wound heals, endothelial cells and myofibroblasts leave behind deposits of the ECM and collagen protein, contributing to tissue repair. Interaction between the dermis and epidermis helps in regulating and restoring the skin’s homeostasis function while maintaining its overall integrity. This period might extend from a few months to a year, depending on the wound [45].

### Electrospun nanofibrous membrane as a dermal substitute

Millions of individuals worldwide are affected by a leading source of morbidity, which is chronic non-healing wounds with extensive injury areas [46]. For the management of such wounds, bioresorbable skin substitutes with optimum biomechanical characteristics and the ability to imitate ECM are required [47]. Currently, the majority of scaffolds are non-bioresorbable. The human skin is made of multiple layers and has a unique geometry [48]. Several skin substitutes are currently available in the wound healing market, and there has recently been great advancement in this sector with the development of many dermo-epidermal substitutes. However, there is still a need for a full-thickness dermal substitute [49]. In this regard, nanotechnology has seen remarkable work in the biomedical field in recent years, offering promising solutions for the development of more effective wound-healing substitutes [50]. Recent advancements in tissue regeneration have further led to the development of innovative wound dressings such as hydrogels and electrospun scaffolds [51].

**Figure 2:**
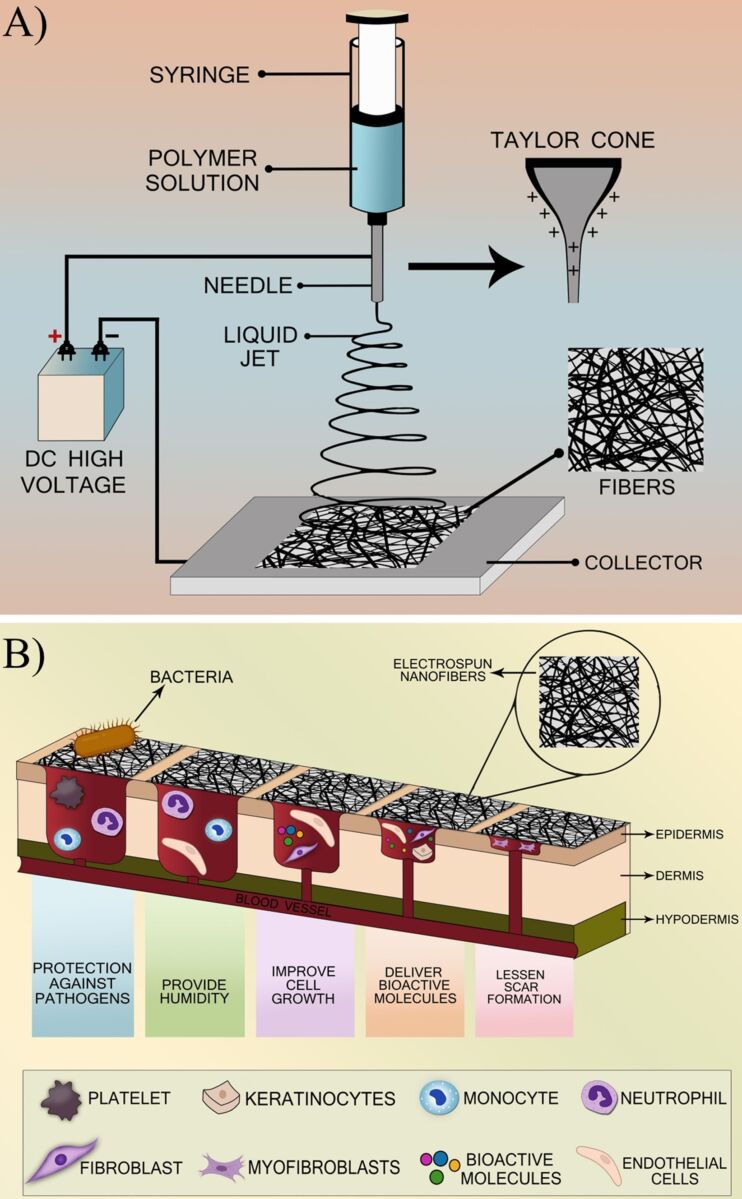
Electrospinning process and the benefits of electrospun nanofibers in wound healing. (A) In electrospinning, a high-voltage electric field is used to create nanofibers from a polymer solution that accumulates on the collector. (B) Significant advantages of these electrospun nanofibers including enhanced tissue regeneration, improved drug delivery, and superior moisture management, which collectively promote faster and more effective healing.

Electrospinning is an extremely flexible technique that can convert liquids, suspensions, or melts into continuous fibers with nano/microscale diameters [52]. It is one of the most extensively used methods for continuous fiber preparation nowadays, which works by using electrostatic forces to produce and stretch fibers from a polymer solution [53]. Typically, a polymer solution is passed through a capillary, and a high voltage is applied to charge the solution’s particles, which produces an attractive force [54]. When the solution’s surface tension is overcome at a critical voltage, a jet shoots out of the capillary’s tip toward a grounded collector (Figure 2A) [55].

The morphology of electrospun polymer fibers is affected by electric field intensity, solution viscosity, charge density of the solution, and solution supply rate [56]. The size of the fibers also affects the performance of electrospun nanofiber composites [57]. Numerous polymers and precursors including polylactic acid, PU, SF, cellulose, and collagen, along with their composites and derivatives can be electrospun [58]. Electrospinning has emerged as a versatile technique investigated across various fields, including tissue engineering, drug delivery, filtration, wound dressings, self-cleaning surfaces, biotechnology, environmental engineering, and green chemistry [59]. This method facilitates the creation of highly porous 3D structures with an extensive surface area and desirable chemical and physical properties, making the resulting nanofibers ideal for applications such as biosensors, antimicrobial surfaces, scaffolds, photocatalysis, and solar energy technologies [60]. Moreover, electrospinning allows for the incorporation of multiple components with distinct morphological characteristics, positioning it as a promising approach for developing innovative materials, particularly in biological contexts [61–62]. The demand for electrospun membranes is rapidly increasing in the medical sector, attributed to their unique surface properties and high porosity [63–64]. Additionally, electrospun membranes can be designed to enhance critical attributes such as biocompatibility, cell attachment, nontoxicity, biomechanical strength, and cellular responses. These membranes can be tailored with a diverse range of fibers, weights, densities, porosities, pore size distributions, chemical compositions, morphologies, hardness levels, and elastic properties [65–66]. Importantly, electrospun membranes and the ECM share substantial structural and functional similarities as both serve as supportive framework. Electrospun membranes are specifically designed to replicate the fibrous architecture and functional properties of the ECM, thereby promoting cellular activity and facilitating tissue regeneration in the same way the natural matrix does within the body [67]. Yildiz et al. used polycaprolactone (PCL)/SF to develop nanofibers as an improved skin substitute for treating chronic wounds or burns. The addition of SF increased tensile strength and Young’s modulus through intermolecular interactions. Results showed that the PCL/SF nanofibers had suitable hydrophilicity for cell proliferation and promoted higher keratinocyte proliferation and viability after seven days compared to pure PCL and SF nanofibers [68]. In another study, Lee et al. explored the use of 3D electrospun SF nanofibers as a dermal substitute for full-thickness skin defects. Although electrospun SF nanofibers are highly biocompatible, their tiny pore size inhibits cell penetration. To solve this, sodium chloride crystals of various sizes were incorporated into the nanofibers during fabrication, resulting in increased pore size. The wound healing properties of the 3D SF scaffold were compared with Matriderm^®^ (dermal substitute). Results showed that the 3D SF scaffold demonstrated similar dermal regeneration, but degraded more efficiently without causing wound contracture, indicating its potential for treating full-thickness wounds [69].

Effective electrospun scaffolds enhance the entire wound-healing process, promoting quick wound recovery by creating suitable conditions for tissue repair [70–71]. As a dermal substitute, electrospun nanofibers offer several advantages compared to conventional woven and non-woven gauzes, particularly in their ability to effectively reduce inflammation, control infection, maintain a moist environment, promote cell growth, deliver bioactive molecules, and reduce scar formation throughout the wound healing process as shown in Figure 2B [72]. Although various materials can be used as effective dermal substitutes, this study focuses on electrospun silk/PU composites, which hold remarkable potential due to the biocompatibility of silk and the mechanical characteristics of PU.

### Silk

**Figure 3:**
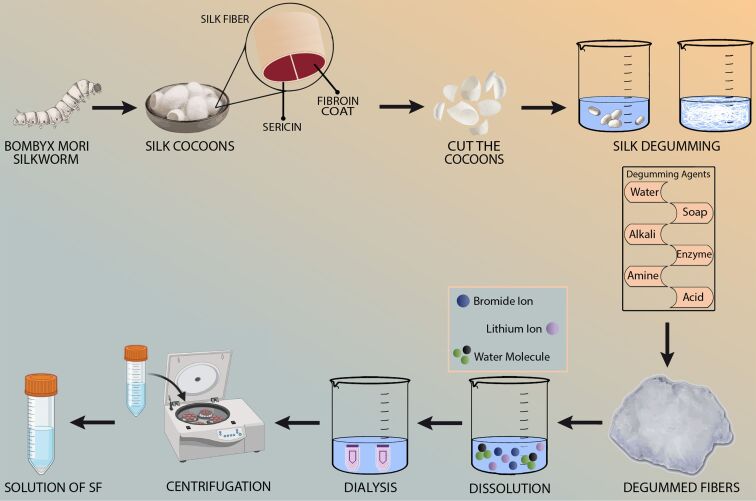
Schematic diagram outlining the extraction process of SF. Created in BioRender. Batool, S. (2025) https://BioRender.com/q23o879. This content is not subject to CC BY 4.0.

The silk from the silkworm *Bombyx mori* (also known as mulberry silk) is the most widely characterized and has been used in biomedical applications for centuries [73]. This silk comprises two proteins called fibroin and sericin. Fibroin is present in the thread core and is responsible for approximately 70% of the total thread weight, while sericin is present on the outside and accounts for roughly 30% of the total silk thread weight [74]. The sericin protein in the silk thread needs to be removed for biomedical applications because it can lead to allergic reactions [75–76]. Recently, it has been reported that sericin, when used alone, is a biocompatible material. However, combining SF and sericin compromises its biocompatibility [77]. The SF produced by spiders does not contain sericin, but its quantity is not sufficient for commercial applications. Hence, the SF biomaterial is usually obtained from silkworms [78]. To obtain SF, cocoons from *Bombyx mori* are first collected and cut into small pieces. The silk is then degummed using several methods such as urea, enzyme, and borax/HCl buffer treatments to effectively remove sericin. After removing the sticky protein, the fibroin fibers are dissolved in an aqueous solution, usually with lithium bromide and similar solvents. Once dissolved, the solution undergoes dialysis to remove any impurities. Finally, the solution is centrifuged to remove any solid particles, resulting in a clear SF solution ready to be electrospun as shown in Figure 3 [79]. Silk contains 30–40% amorphous and 60–70% crystalline regions; crystalline silk is composed of silk I (α-helical), silk II (β-sheets), and silk III (hexagonal crystalline), whereas random globules make up the amorphous region [80]. Silk I with α-helical structure can be turned into silk II with β-sheets through shearing, spinning, heating, or by using methanol or ethanol solvents; this transition is considered irreversible [81]. Triple helices of collagen and β-sheets are found in various silks [82]. These types of proteins often exhibit remarkable mechanical characteristics, in contrast to globular proteins. SF nanofibrils are the building blocks of SF filament and have a diameter of around 3.5 nm. These nanofibrils originate from SF microfibrils, which typically range from 200 to 300 nm [74]. The basic structure of SF is formed by the HL complex, in which H is the heavy peptide chain with a molecular weight of 350 kDa and L is the light peptide chain of about 25 kDa; both are bonded together by disulfide connections [83]. The glycoprotein P25 is non-covalently connected to the HL complex, and it provides integrity to the fibroin structure. The ratio of H fibroin, L fibroin, and p25 in mulberry silk is 6:6:1 [84].

In vivo studies showed that SF scaffolds promote better wound healing and skin regeneration than porcine dermis, hydrocolloid dressings, or dermal matrix [85]. In vitro studies showed that the use of electrospun silk nanofibers favors the attachment of human fibroblasts, keratinocytes, and deposition of collagen type I [86]. Silk can be processed into hydrogels, scaffolds, and films with the incorporation of bioactive factors such as growth factors, drugs, and stem cells to facilitate the process of growth and regeneration [87]. Fitzpatrick et al. develop a scaffold by combining silk–hydroxyapatite bone cement with growth factors such as bone morphogenetic protein-2 (BMP2), vascular endothelial growth factor (VEGF), and neural growth factor (NGF) to promote bone regeneration, vascularization, and nerve integration. The scaffolds were designed with precise porosity and geometry to promote new bone growth while mimicking the structure of natural bone. The scaffold’s mechanical properties were appropriate for bone tissue application, and the scaffold was cytocompatible and osteoconductive. In vitro studies showed that the scaffold successfully supported the osteogenic differentiation of human mesenchymal stem cells, migration and proliferation of endothelial cells, and growth of neural stem cells. The combination of BMP2, VEGF, and NGF improved osteoblastic differentiation as evidenced by the upregulation of specific osteogenic genes [88].

Zhou et al. improved bone tissue engineering by creating a composite biomimetic scaffold incorporating autologous concentrated growth factor (CGF) to repair bone defects. Freeze drying and chemical cross-linking were used to develop a silk fibroin/chitosan/nanohydroxyapatite (SF/CS/nHA) scaffold in three different concentrations (3%, 4%, and 5%). The 4% SF/CS/nHA scaffold was shown to be the most effective for bone repair. In vitro studies involved seeding bone marrow mesenchymal stem cells (BMSCs) on these scaffolds and adding CGF. The results demonstrated that the SF/CS/nHA scaffold combined with CGF promoted better cell adhesion, proliferation, and osteogenic differentiation of BMSCs than other groups. In vivo, a rabbit model with major bone defects was used to assess the scaffold’s efficiency. In imaging and histological tests, the SF/CS/nHA-BMSC scaffold combined with CGF significantly improved bone repair compared to the control groups [89]. Safdari et al. incorporated ceftazimide (STZ) to develop antibiotic-loaded SF/gelatin nanofibers through electrospinning for wound dressing applications without affecting the structure or bioactivity. The effective encapsulation of STZ was confirmed by FTIR, and the nanofibers showed high cytocompatibility in cell viability tests. STZ was released from nanofibers over 6 h, and its antibacterial activity was demonstrated through the formation of a zone inhibition in *Pseudomonas aeruginosa* cultures. The findings suggest that STZ-loaded SF/gelatin nanofibers are highly effective against post-surgical infections and promote wound healing, particularly in cases where traditional oral and injected antibiotics are ineffective [90]. Millán-Rivero et al. explored the usage of an electrospun SF scaffold cellularized with human Wharton’s jelly mesenchymal stem cells (Wj-MSCs) in a murine excisional wound splinting model. The finding showed that Wj-MSCs on SF scaffolds improved wound healing by increasing neoangiogenesis and re-epithelization while reducing inflammation and fibrotic scar formation. These results were due to the presence of CD90-positive cells in the treated wounds, which helped to produce well-vascularized granulation tissue and improved tissue regeneration compared to single treatments [91].

The structure, fabrication, processing, and functionalization of SF facilitate its usage in optics as a biosensor and implantable device [92]. Malinowski et al. showed that conventional transparent films often lack adequate haze, but SF films exhibit ultrahigh optical transparency (>93%) and transmission haze (>65%). In this study, soft lithography was used to create and analyze SF films with various nanostructures that exhibit tunable optical properties depending on the structure. When combined with silicon photodiodes, the power conversion efficiency increased by 6.96% with flat SF films and 14.9% with nanopatterned SF films. This demonstrates the ability of SF films to enhance light trapping in photoelectronic devices. Furthermore, combining SF films with biodegradable solar cells has the potential to power next-generation biomedical equipment, providing long-term energy solutions for diagnostic and therapeutic applications [93].

#### Silk fibroin-based electrospun fibers for biomedical applications

**Figure 4:**
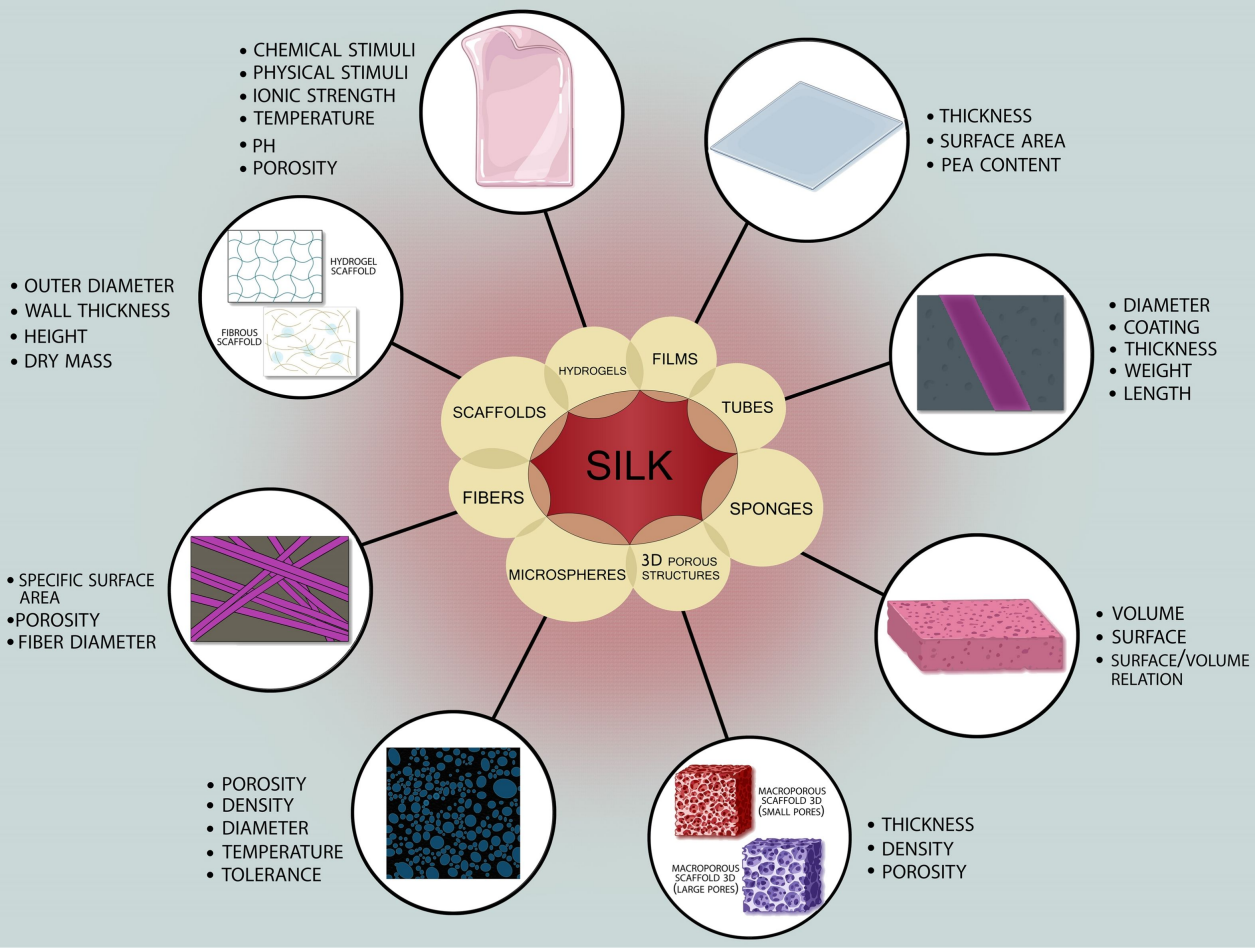
Biomedical applications of electrospun SF, highlighting the key parameters that influence its performance. Created in BioRender. Batool, S. (2025) https://BioRender.com/w01p597. This content is not subject to CC BY 4.0.

Silk from *Bombyx mori* has been used as biomedical suture for centuries [94]. Generally, silks are protein polymers that are spun into fibers, which provides a wide range of material options for controlled release systems, biomaterials, and tissue engineering scaffolds [95]. An essential basis for using these natural proteins for biomedical applications is their relative environmental stability in contrast to globular proteins, along with their options for gene-customizing sequences [96]. SF has been extensively utilized in designing matrix scaffolds for tissue engineering, particularly for bone, ligaments, tendons, blood vessels, and cartilage, where mechanical properties and biological interactions are crucial. SF can be fabricated into foams, films, fibers, meshes, and hydrogels, as shown in Figure 4 [97]. Films made of fibroin and collagen were comparable to those made of sericin in their ability to promote cell attachment, physiological morphology, and growth. These findings support the idea that fibroin extracted with sericin is a suitable matrix for cell and tissue growth [98]. Silk implants with slower degradation rates or those placed in soft tissues often provoke a stronger immune response than implants that degrade quickly or are used in hard tissues. The physiological response to implanted silk material depends on several factors, including the material’s format, degradation rate, and implantation site [99].

In vitro studies showed that various proteases, such as protease K, collagenase, and α-chymotrypsin can degrade silk, resulting in a gradual reduction in material strength and weight over time [100]. In vivo, silk degradation is also affected by the β-sheet content; scaffolds that have a high concentration of β-sheets exhibit slower degradation [101]. Additionally, there is a direct correlation between immune cell infiltration and silk degradation. Although other cell types may contribute to silk degradation, immune cells are primarily responsible for this process [102]. Silk can be modified, allowing for cell adhesion and proliferation, which eventually causes the scaffolds to degrade and be replaced by natural tissue. Many silk scaffolds used in tissue engineering are designed with high porosity and provide a platform for the cells to self-assemble into vessel-like structures [103–104]. When constructing tissue models using permeable freeze-dried silk, a highly porous sponge with an adequate pore size is crucial for controlling cell migration and proliferation, particularly when the objective is to generate a dermally well-vascularized layer [105]. The most common approach for constructing a double-layer skin is using a porous matrix in which fibroblasts fill the silk framework before keratinocytes are placed inside and allowed to mature [106].

In 2019, Sofregen Medical, Inc. gained FDA clearance for Silk Voice^®^, a scaffold composed of renewed or solubilized silk protein. SF offers fundamental qualities for cutaneous wound healing, including its ability to interact with fibrin and blood platelets, contributing to its hemostatic properties [102]. Biswas et al. developed an SF fiber-based hemostatic agent of alkaline-hydrolyzed silk microfibers (AHSMs) from *Bombyx mori* silk. Sodium hydroxide was used to randomly chop the microfibers. In vitro tests using goat and human blood revealed a substantial decrease in clotting time, prothrombin time, and activated partial thromboplastin time compared to untreated silk fibers. This study suggests that AHSMs could be a cost-effective and efficient hemostatic agent for trauma treatment [107].

Patil et al. explored three types of silk forms, namely, regular film, lamellar porous film, and electrospun silk nanofibers for wound dressings. Factors such as solvent solution, electric field intensity, spinning distance, and SF concentration affected the properties of the SF nanofibers. Findings showed that if the concentration of SF is low, the produced SF mats often exhibit clustered or beaded fibers. Such poor molecular and fibril alignments are responsible for poor mechanical properties of SF electrospun nanofibers [85]. The discharge absorption capability of SF increased hydration and moisture-holding ability [108]. The physical durability of SF supports the strength of wound dressing, inhibits disruption of the wound bed, and allows for adaptation to size and form of a wound [104]. The inherent bioactive properties of SF, which also stimulate cell movement and tissue regeneration, allow for neovascularization, accelerated re-epithelialization, and tissue development [109]. Without the need for a cross-linker, SF-based composites can be customized with a range of antimicrobial drugs, growth stimulants, and other bioactive substances [110]. Ren et al. developed a micro–nanofiber dressing by electrospinning a blend of SF, chitosan, and halloysite nanotubes (HNTs) loaded with the antibacterial agent chlorhexidine digluconate (CHD). The addition of HNTs considerably altered the nanofiber structure, and increased the material’s thermal stability and mechanical capabilities, while improving blood clotting as demonstrated by reduced whole blood coagulation time. This study also indicated a sustained release of CHD over 8 h, with a faster drug release rate in an acidic environment. These properties highlight the dressing’s potential for effective hemostasis and infection control in wound healing [111].

Silk as a cost-effective natural material in its raw form simplifies the commercialization of *Bombyx mori* SF products. The FDA has granted 510k clearance for the use of SERI^®^ (surgical scaffold) and regenerated silk-based scaffolds [112]. Aside from its historical use in wound dressing, the development of skin grafts represents a distinct application of silk-based technology. Clinically, silk-based matrices can be used to create dermoepidermal bio-artificial skin grafts and dressings, with significant potential for future applications in plastic surgery [113].

### Polyurethane

The biocompatibility of polyurethane (PU) makes it well suited for interactions with biological fluids and tissues [114]. Its ability to imitate the mechanical characteristics of natural tissues further enhances its appeal in biomedical applications [115]. The surface of PU can also be modified to improve cell adhesion and proliferation, facilitating its integration with biological systems [116]. Moreover, PU can be tailored to have particular mechanical characteristics and controlled degradation rates, allowing it to be adapted to various biological environments [117]. Its widespread availability and ease of processing make it an attractive option for developing a wide range of medical devices and implants [118]. PU is typically synthesized from three key components, that is, polyol, cross-linker, and diisocyanate. These components form the urethane link (–NHCOO–), which is the fundamental component of PU [119]. There are more than 500 types of polyols that are commercially available, such as polyether, polyester, polycaprolactone, and polycarbonate polyols. The polyol determines the physiochemical properties of PU [120]. The isocyanate can be aliphatic, dicycloaliphatic, cycloaliphatic, aromatic, or polycyclic. Compared to aliphatic isocyanates, aromatic isocyanates are more reactive and used in the preparation of rigid PU [121–122]. Short-chain diols are generally used as chain extenders, and they affect the properties of hard segments [123]. Sometimes, catalysts are also used to speed up the PU synthesis. The initial ratio of the three key components and synthesis route and parameters are the primary factors that influence the properties of PU, such as degradability, flexibility, and hydrophilicity. By adjusting these factors, the characteristics of PU can be effectively controlled [124]. Urethane, a derivative of carbamic acid, serves as a repeating unit in PU and typically exists in ester form [125]. For over 20 years, PU has been utilized in the human body, becoming one of the most extensively studied materials for biomedical applications. Versatility and adaptability of PU make it a key compound in scientific research, excelling many other materials in this field [126].

#### Biostable polyurethane compounds

Biostability is the key requirement for the prolonged functionality of implantable biomaterials. PU is prone to degradation by oxidative species released by macrophages and foreign bodies [127]. The degree of crystallinity is the key factor in developing biostable PU compounds. PU has two segmented structures, that is, a hard segment (made from isocyanate) and a soft segment (made from diols) [128]. A higher hard segment content will improve the hydrolytic stability and give greater resistance against enzymatic hydrolysis [129]. Moreover, it promotes the formation of more hydrogen linkages that give greater biostability to PU-based biomaterials [130]. Polyether- and polyester-based PUs are prone to oxidation and hydrolysis, while polycarbonate-based PUs show better biostability [131]. The biostability of silicone-based PUs is even higher [132]. The improved tear strength, tensile strength, and biostability make them promising materials for biomedical applications. There are only few materials suitable for long-term implants, with elastomer-based soft tissue interface materials being particularly rare. Currently, silicone rubber and, to a lesser extent, PU are the only elastomeric compounds that have demonstrated potential for such applications [133]. Aromatic PUs seem to be more stable than aliphatic PUs. PUs with no or few ether linkages have given rise to a new class of PUs known as “biostable” [134].

Wheatley et al. reported that sheep with a non-biostable PU mitral heart valve prosthesis survived for up to six months. However, issues such as surface degradation and accumulation of calcified fibrin/thrombus compromised leaflet mobility and hydrodynamic performance. Newly accessible biostable PUs have shown promise in addressing these issues. After six months of implantation, biostable PUs improved blood compatibility, preserved leaflet flexibility, and maintained regular valve performance. This suggests that the biostable PUs could enhance the longevity and performance of artificial heart valve prostheses [135]. Several studies focused on creating novel PU compounds with constituents that are less susceptible to oxidative breakdown after realizing the necessity of PU with improved biostability after long-term implantation, especially regarding the soft grades (low modulus and high terminal elongation) [136–138]. Many families of PU with increased oxidative stability and customizable mechanical and physical properties were developed in the 1980s and 1990s. Polyols with a hydrocarbon core are appealing for developing PUs with higher oxidative and hydrolytic stability, and this is one of the primary methods used to produce biostable PUs. PUs are made biostable by increasing their crystallinity. PU remains valuable in biomedical applications because of its elastomeric characteristics, lubricity, superior abrasion resistance, and high tensile strength [139].

#### Biodegradable polyurethanes

The ease of manufacturing of PU and its tunable elastic, mechanical, and biodegradable properties make it an ideal material for wound healing, tissue engineering, and drug delivery applications [140]. Medical implants such as vascular grafts and cardiac pacemakers can be made of PU. However, PU in such devices may degrade when placed within the human body, which could lead to device failure [141]. Many researchers have found it difficult to explore these events because of the complexity in vivo. Hence, many studies have been conducted to learn more about the ways PU degrades in living beings [142].

Several modifications can be made to PU to make it an excellent material for wound healing and skin substitute by blending, surface functionalization, and adding active moieties. PU with hydrophilic groups and long chains forms fewer crystals, making it more biodegradable [124]. Degradation is limited in crystalline regions, while amorphous regions get degraded easily within PU. The molecular structure and composition of the polymer, its molecular weight, crystallinity, and the presence of cross-links and additives are a few elements that impact polymer degradation kinetics. The degradation is also affected by surface-modifying macromolecules (SMMs). A variety of SMMs resists enzymatic action. Some SMM formulations are incompatible with PU, which can increase biodegradability. For the medical field, biodegradable polyester PU is derived from polycaprolactone diols [143]. Three mechanisms, namely, hydrolysis, enzymatic degradation, and oxidation, are primarily responsible for the extracellular breakdown of synthetic biodegradable polymeric materials in living organisms. Certain aliphatic polyesters are undeniably biodegradable and biocompatible polymers. It is possible to convert non-biodegradable polymers into biodegradable ones using a combination of low molecular mass compounds or degradative radiation [144].

There are several issues regarding implantable PU, including inadequate long-term molecular strength in vivo and susceptibility to calcification. The molecular instability of PU is challenging for permanent implants, yet it might be purposefully used to create elastomeric biodegradable biomaterials. Polyester-based PUs have low hydrolytic stability and are, therefore, unsuitable for long-term implanted devices. Nonetheless, PUs are a class of polymers that have good biocompatibility. PUs provide significant potential for modifying polymer structures to obtain various mechanical properties [145].

#### Water-based polyurethanes

The development of water-based polyurethanes (WPUs) has revolutionized the field of PU synthesis [146]. It has offered a greener and environmentally friendly PU synthesis route by replacing the common process of solvent-based PU synthesis. The emission of volatile organic compounds in synthesis and application can be avoided by adopting water-based PUs [147]. This has brought a shift in urethane research. WPUs have emerged as a new area for exploring an environmentally friendly variant of urethanes. Typical 3D printing frequently uses heat, organic solvents, or cross-linkers, which decrease the bioactivity of the chemicals. It may be challenging to include bioactive compounds for controlled release. Thus, a water-based biodegradable PU has been utilized to develop 3D printing materials with regulated bioactivity for cartilage tissue engineering [148]. The printing ink contains a water-based dispersion of synthetic biodegradable PU nanoparticles, hyaluronan, and bioactive substances. The ink was used at low temperatures to produce conforming scaffolds. With the timely release of bioactive components, these scaffolds encourage the self-aggregation of mesenchymal stem cells and induce chondrogenic differentiation of mesenchymal stem cells and the production of the matrix for cartilage reconstruction [149]. Other than cartilage tissues, 3D-printed WPU scaffolds can be customized to be used for various connective tissue engineering applications, such as loose connective tissues, dense fibrous connective tissues, skeletal tissues, adipose tissues, and elastic connective tissues with controlled bioactivity.

#### Electrospun polyurethane fibers for biomedical applications

PU comes in various forms, including flexible foams, coatings, adhesives, and rigid thermosets [150]. PU is frequently used as a material for constructing nanowebs because of its chemical stability, diffusion coefficients, superior mechanical capabilities, and outstanding nanofiber-forming characteristics [151]. Semipermeable dressings, many of which are composed of PU, are claimed to speed up wound healing [152]. Using melt electrospinning, degradable and biocompatible aliphatic PUs can also be created into scaffolds [153]. The qualities of PUs can range widely depending on their structure [154]. One of the key benefits of PU over all other polymers is the ability to adjust the structure as the material is being processed. In the electrospinning process, a variety of PUs was employed; some were produced beforehand for the researchers’ intended usage, while others were used just as they were provided [155]. Because of its excellent barrier qualities and oxygen permeability, PU is commonly utilized in wound dressings [156]. Since PU is soft and hydrophobic, using it as a dressing material on its own would be unsuitable because the dressing might be too soft for use in the medical field. Also, its hydrophobic property might prevent fluid from seeping from the wound surface [157]. To investigate PU composites for the possible use in wound dressings, researchers have carried out a considerable amount of research. To obtain desired features like higher cell survival, cell adhesion and proliferation, increased blood clotting capacity, and better hydrophilicity, PU was employed as the base polymer and combined with CA and zein (a natural polymer) [158]. Numerous studies have shown that manufacturing PU with organic polymers, such as chitosan, helps to create a synergistic composite. A wide range of composite materials, with both chitosan and PU, have been thoroughly studied. The findings show that these two materials work well together, each making up for the deficiencies of the other material [159–160]. PU-based scaffolds were also investigated as a platform for neuronal development and embryonic stem cell culturing [161]. For biological applications, electrospun PU nanofibers including antibacterial substances such as silver nanoparticles, 4-vinylpyridine, or streptomycin sulfate were also created. These researches combined the use of wound dressings with the antibacterial properties of PU nanofibrous membranes [162].

PU/gelatin and PU/cobalt nitrate were used to create mixed nanofibers scaffolds used to develop wound dressings [162–163]. It was shown that cobalt nitrate added to the PU wound dressing resulted in improved physicochemical properties, blood compatibility, and fibroblast proliferation parameters [163]. One-layer wound dressings cannot meet all therapeutic demands because of their distinct features and limitations [164]. Therefore, there has been a lot of interest in bilayer wound dressings consisting of two layers, each with distinct characteristics [165]. A dense membrane made of PU and an ethanolic extract of propolis (EEP) was electrospun with a polycaprolactone/gelatin (PCL/Gel) scaffold. The PCL/Gel scaffold was employed as the sublayer to promote cell adhesion and proliferation, while the PU/EEP membrane was utilized as the top layer to shield the wound region from external harm and dehydration. Microstructure, mechanical characteristics, surface wettability, anti-microbial action, biodegradability, biocompatibility, and effectiveness of the bilayer wound dressing were also examined [166].

### Light-activable nanostructures

Biomaterials that respond to light are novel technologies in medicine. They are administered to improve the healing of wounds by responding to particular light wavelengths. Such sophisticated materials are enabled by micro- and nanoscale structures that enhance their tissue interactions and provide more focused therapeutic treatment [167]. These materials are embedded with responsive elements like nanoparticles that capture light in the near-infrared (NIR) or visible range and transform it to energy or heat to elicit specific responses. Such materials can, upon light exposure, release therapeutic payloads in the form of antimicrobial or anti-inflammatory drugs, heat up to a temperature that destroys bacteria, or actively stimulate tissue repair [168]. Treatments can be modulated by changing the type, intensity, and duration of light, and specific attention can be given to the wound with minimal interference with the surrounding tissues. This has many advantages in wound healing such as reduced infection risks, promotion of new skin cells, blood vessel formation, and minimum scarring. These biomaterials also show increased effectiveness regarding cell attachment and proliferation because the nanostructured design offers a large surface area. They are biocompatible, lowering the possibility of negative immune responses, and are adaptable to the use as films, gels, or scaffolds for different kinds of wounds [169]. Materials such as gold nanoparticles, graphene oxide, and silicon nanostructures are frequently utilized owing to their photothermal properties with drug-release capability, which can result in sustained drug delivery, cell attachment, and antibacterial action. Particularly noteworthy is the fact that these materials are activated by light in a non-invasive manner, which greatly reduces the discomfort of the patients during the procedure [170].

Besides wound healing, light-activated nanostructured biomaterials have promising applications in treating neural disorders. For example, in nerve tissue engineering, light-responsive scaffolds and nanomaterials facilitate neural stem cell proliferation and differentiation to repair damaged neural tissue [171]. They are also widely used for cancer treatments such as photothermal and photodynamic therapy, where light activation creates heat or reactive oxygen species to severely damage and kill cancer cells while reducing the harm done to healthy surrounding tissues [172]. In addition, they are also vital in advanced drug delivery systems where highly specific and localized release of drugs can be achieved through light, thus increasing the efficiency of various therapies [173]. Rybak et al. formulated a new 3D-printed hydrogel wound dressing for infected wounds. This composite contains short filaments of electrospun poly(lactic-*co*-glycolic) acid (PLGA) fibers embedded with gold nanorods (AuNRs) and the anti-inflammatory drug dexamethasone. The filaments are incorporated in a gelatin methacrylate/sodium alginate hydrogel scaffold. The AuNRs render the material responsive to NIR light for controlled heating and drug release to eliminate bacteria. Furthermore, the hydrogel is highly antibacterial, with tested efficacy against *Staphylococcus aureus* and *Escherichia coli* of more than 99.9%. It also enables cell proliferation, suppresses inflammation, stimulates angiogenesis, and facilitates tissue regeneration. Additionally, the scaffold is flexible, can be conformed to the site of the wound, and shows good water retention and high mechanical strength [174].

#### Light-activable SF/PU nanostructures

To repair damaged nerves, Hu et al. produced particles that can be classified as gold–polydopamine blackspheres (AuPBs), capable of absorbing NIR-II light and converting it into heat. These particles were incorporated into an electrospun film by combining poly(ʟ-lactic acid) (PLLA) with SF to obtain a PLLA-SF parallel films. The films had high strength and hydrophilic properties making them suitable for medical applications. The films were then shaped into a scaffold for implants in nerve injury sites. The scaffold was implanted at the site of sciatic nerve injury more than 1 cm deep in the skin. The polydopamine present in the AuPBs reduced inflammation around the injury and helped increase the production of VEGF, which aids in vasculogenesis. At the same time, the heat produced by NIR-II light activated specific ion channels of the nerve cells. These channels were permeable to calcium, and the influx of this ion triggered the release of important growth factors such as brain-derived neurotrophic factor and NGF. These growth factors increased the alignment and myelination of Schwann cells, which are critical for nerve regeneration and repair. The study also showed that the AuPBs were cell-compatible, which meant they were non-toxic and supported cell growth [175].

Zhou et al. focused on the fabrication of a novel composite membrane suitable for photothermal cancer therapy based on black phosphorus (BP) nanosheets because of their high biocompatibility and photothermal efficacy. SF was used as an exfoliating agent in stable liquid exfoliation with ultrasound to incorporate BP nanosheets for improved stability and processability. As a result, silk fibroin-doped black phosphorus nanosheets (BP@SF) were produced, which possess good water dispersity and long-term stability. BP@SF nanosheets were consolidated into fibrous membranes based on SF and PLGA by electrospinning. The SF/PLGA/BP@SF membranes had smooth and uniform fibers, high tensile strength, and good photothermal properties. Moreover, when exposed to NIR light, the temperature of the membranes raised; the photothermal performance was tunable depending on the amount of BP@SF incorporated and the NIR power. These membranes were shown to ablate HepG2 cancer cells in vitro, revealing the prospect for localized cancer treatment [176]. Lv et al. created a new light-sensitive shape memory polyurethane (SMPU) by using micro/nanofibers of polydopamine (PDA)-coated poly(ε-caprolactone). The aim of this method was to provide a solution to the problems of traditional heat-induced shape memory polymers. Additionally, PDA was chosen as it is highly adhesive, biocompatible, and a great photothermal converter. SMPU fibers were made using electrospinning, and the fibers were pre-coated with PDA using post-synthesis immersion. The results indicated maintenance of shape memory characteristics of the fibers under light stimulus. Notably, through thicker photothermal coatings or higher light intensities, equilibrium surface temperatures were elevated without compromising the performance of the shape memory. To determine the biocompatibility of the fibers, BMSCs were used. The results disclosed that adhesion, viability, and proliferation of the cells were enhanced by the PDA coating. The bioactivity and wettability of the coatings increased the chances of osteoblast adhesion and survival, proposing that the PDA/SMPU fibers have great potential [177].

### Silk fibroin/polyurethane-based composites

When PU and SF are electrospun, they form composites with unique qualities, including biodegradability, increased mechanical strength, and flexibility. These composites makes the development of advanced wound dressings, scaffolds for tissue engineering, and drug delivery systems possible. These developments are essential for adjusting the composites’ biological and physical characteristics, which will enhance patient outcomes [178]. Researchers have thoroughly investigated the potential of electrospun composites based on SF and PU. To enhance the mechanical characteristics and biocompatibility of the composites, researchers have experimented with various ratios and combinations of SF and PU. In a study, Jiang et al. developed composite nanofibrous mats through electrospinning silk fibroin powder (SFP) and PU blends. They adjusted the electrospinning parameters, such as solvent ratios and polymer concentration, to produce homogenous PU nanofibers. The PU solution was blended with varying concentrations of SFP to create composite nanofibers that had enhanced water permeability, hydrophilicity, and mechanical characteristics. The developed SPU/PU nanofibers have potential uses in environmental studies, separation, and the biomedical area in particular [179].

**Figure 5:**
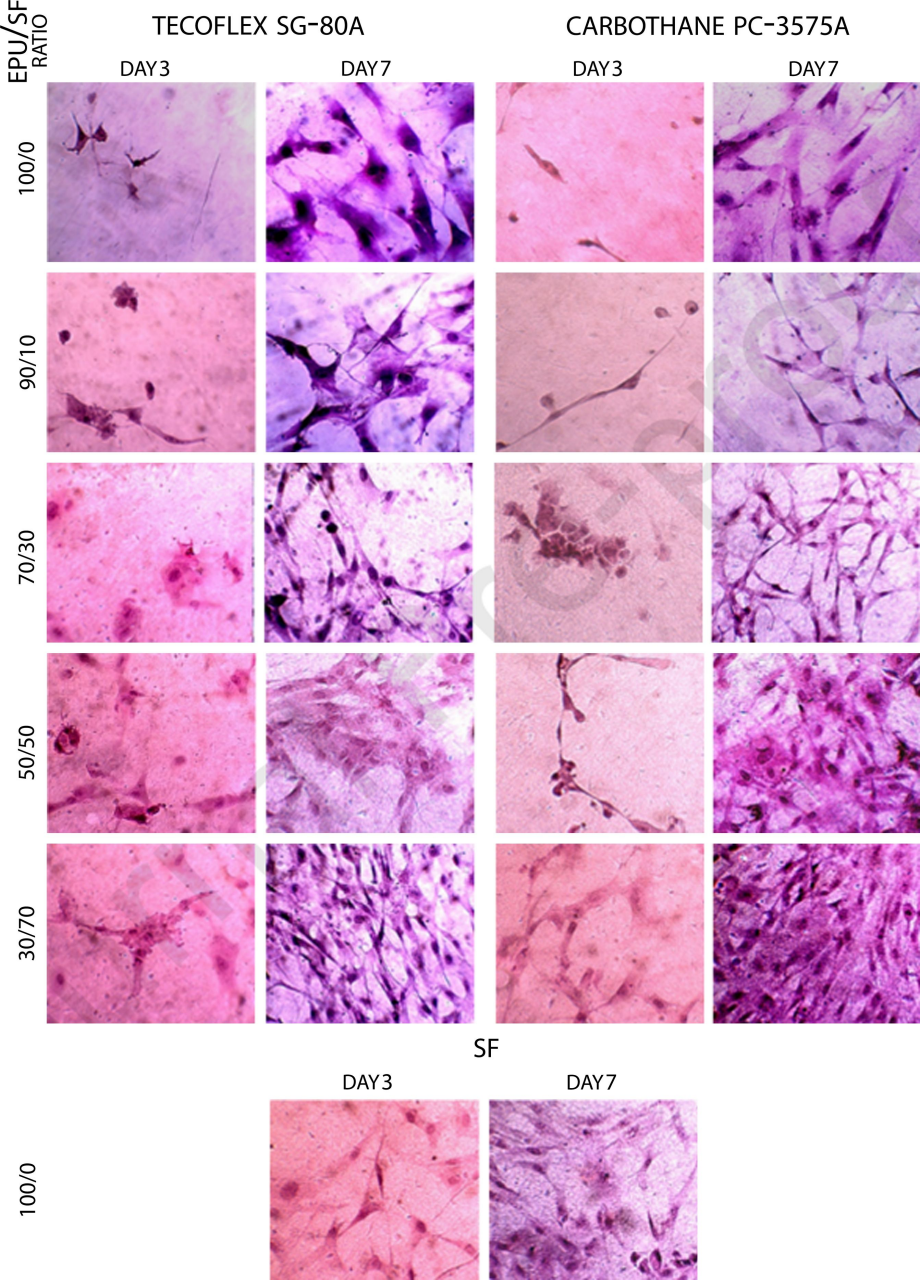
YhFF#8 cells cultured on a scaffold for three or seven days. The EPU/SF 30/70 scaffolds in both EPUs had the maximum cell proliferation, particularly in the case of PC-3575A. Reprinted from [180], European Polymer Journal, vol. 121, by N. Dehghan-Manshadi; S. Fattahi; M. Hadizadeh; H. Nikukar; S. M. Moshtaghioun; B. Aflatoonian, “The influence of elastomeric polyurethane type and ratio on the physicochemical properties of electrospun polyurethane/silk fibroin hybrid nanofibers as potential scaffolds for soft and hard tissue engineering,” article number 109294, Copyright (2019), with permission from Elsevier. This content is not subject to CC BY 4.0.

Yu et al*.* created small-diameter vascular grafts with a structure mimicking natural elastic tissues using a unique electrospinning process. Thermoplastic polyurethane (TPU) and SF were combined at different weight ratios to create the hybrid vascular grafts. Material compositions and solvents impacted the electrospun fiber morphology, and all hybrid grafts showed mechanical characteristics similar to those of normal blood vessels. The wavy fibers produced by the specialized electrospinning collector improved the extensibility of the grafts. Cell culture tests revealed that the hybrid grafts enabled higher cell proliferation than pure TPU grafts, suggesting they could be more biocompatible. Overall, the results demonstrate the potential of these hybrid vascular grafts for the use in tissue engineering [181]. Shrestha et al. developed a fibrous scaffold composed of SF, PU, and functionalized multiwalled carbon nanotubes (fMWCNTs) by electrospinning. Because of its superior mechanical qualities, hydrophilicity, biodegradability, and biocompatibility, the designed PU/SF-fMWCNTs biomaterial is promising regarding nerve tissue engineering. After the scaffold’s shape and structure were examined using various methods, it became clear that fMWCNTs improved ECM absorption and electrical conductivity. In vitro tests showed that the aligned scaffolds significantly increased the development and proliferation of Schwann cells (S42), differentiation, and neurite growth of rat pheochromocytoma (PC12) cells along the fibers. Immunocytochemistry and qRT-PCR demonstrated that the conductive PU/SF-fMWCNTs scaffold increased neuronal expression and axonal regeneration. These findings indicate that the fMWCNTs-based scaffold represents a novel technique for building nerve-guided conduits, emphasizing its potential for peripheral nerve repair in preclinical applications [182]. Dehghan-Manshadi et al. used electrospinning to create hybrid fibrous scaffolds composed of SF and two aliphatic elastomeric polyurethane (EPU) types. They combined EPU and SF in different mass ratios and used various analytical methods to assess the scaffolds’ mechanical and physical characteristics. After seeding fibroblast cells onto various nanofibrous scaffolds, the study discovered that scaffolds containing more SF improved cell adherence and proliferation. The best cell proliferation was seen on the EPU/SF 30/70 scaffold, which had better growth and cell adhesion because of its ideal fiber diameter and mechanical characteristics, as shown in Figure 5. These results imply that the EPU/SF hybrid scaffolds are potential candidates for a variety of tissue engineering applications, such as skin, liver, vascular, and nerve regeneration, because of their customizable features [180].

In another study, Watcharajittanont et al. developed layer-by-layer electrospun membranes using PU and SF, imitating oral soft tissue to facilitate guided bone regeneration. Sequential electrospinning of PU and SF solutions resulted in multilayered membranes with different SF thicknesses. After assessing the membranes’ physical and biological characteristics, it became clear that the membrane with a thin SF core demonstrated enhanced biological performance and appropriate mechanical characteristics, making it a viable option for applications involving guided bone regeneration [183]. Khan et al*.* enhanced the characteristics of PU fibers, which are often too hydrophobic to be used in tissue engineering. Using electrospinning, they developed innovative fibers coated with silver nanoparticles (Ag NPs) to provide antibacterial properties and SF to enhance biocompatibility with biological tissues. Tests revealed that SF and Ag NPs were effectively introduced, and the fibers kept their form. The fibers were considerably less hydrophobic as the water contact angle decreased and moisture absorption rose. Another essential component of bone tissue, hydroxyapatite, was similarly encouraged to develop by the treated fibers. The fibers exhibited potent antibacterial action, successfully preventing *E. coli* and *S. aureus* from growing. Furthermore, HEK 293T cells grew better on the novel composite scaffolds than on untreated PU fibers, with the highest results achieved on scaffolds containing 10% Ag NPs. This study suggests that these novel PU fibers have the potential to aid in wound healing. However, further research is needed to optimize the manufacturing process, test additional healing agents, and conduct animal trials to validate their efficacy [184].

Almasi-Jaf et al. developed a biocompatible heparinized bilayer vascular graft with small diameter. Heparin is a potent anticoagulant, which has been utilized to alter the surface of polymeric materials to increase their compatibility with blood. Excellent mechanical qualities, such as ultimate stress, Young's modulus, and suture retention, were given by the inner layer composed of co-electrospun fibers of heparinized PCL, gelatin, and PU. To obtain a high porosity, which is required for smooth muscle cell activity, the outer layer consisted of a hydrogel composed of chitosan, gelatin, and SF that had been freeze-dried and cross-linked using *N*-hydroxysuccinimide and ethylcarbodiimide hydrochloride. The graft showed strong anti-thrombogenic qualities, a 70% continuous release of heparin over four weeks, and high cell viability for smooth muscle and endothelial cells, suggesting its potential for vascular graft regeneration [185]. Khan et al. developed novel scaffolds for bone tissue engineering with the use of PU micro- and nanofibers. These fibers were mixed with calcite to increase the bone growth-inducing potential and coated with SF and copper nanoparticles (Cu NPs) to further improve their properties. The incorporation of calcite resulted in increased fiber diameter and strength, while the SF coating increased the hydrophilicity of the scaffolds. Also, Cu NPs are known to provide strong antibacterial activity against pathogenic bacteria such as *E. coli* and *S. aureus*. The scaffolds also exhibited remarkable thermal stability, controlled enzymatic degradation, and high cell viability, which enhance cell attachment and proliferation. These characteristics make the scaffolds adequate for bone tissue engineering, fulfilling the mechanical, biological, and antibacterial requirements for a single scaffold material [186].

**Figure 6:**
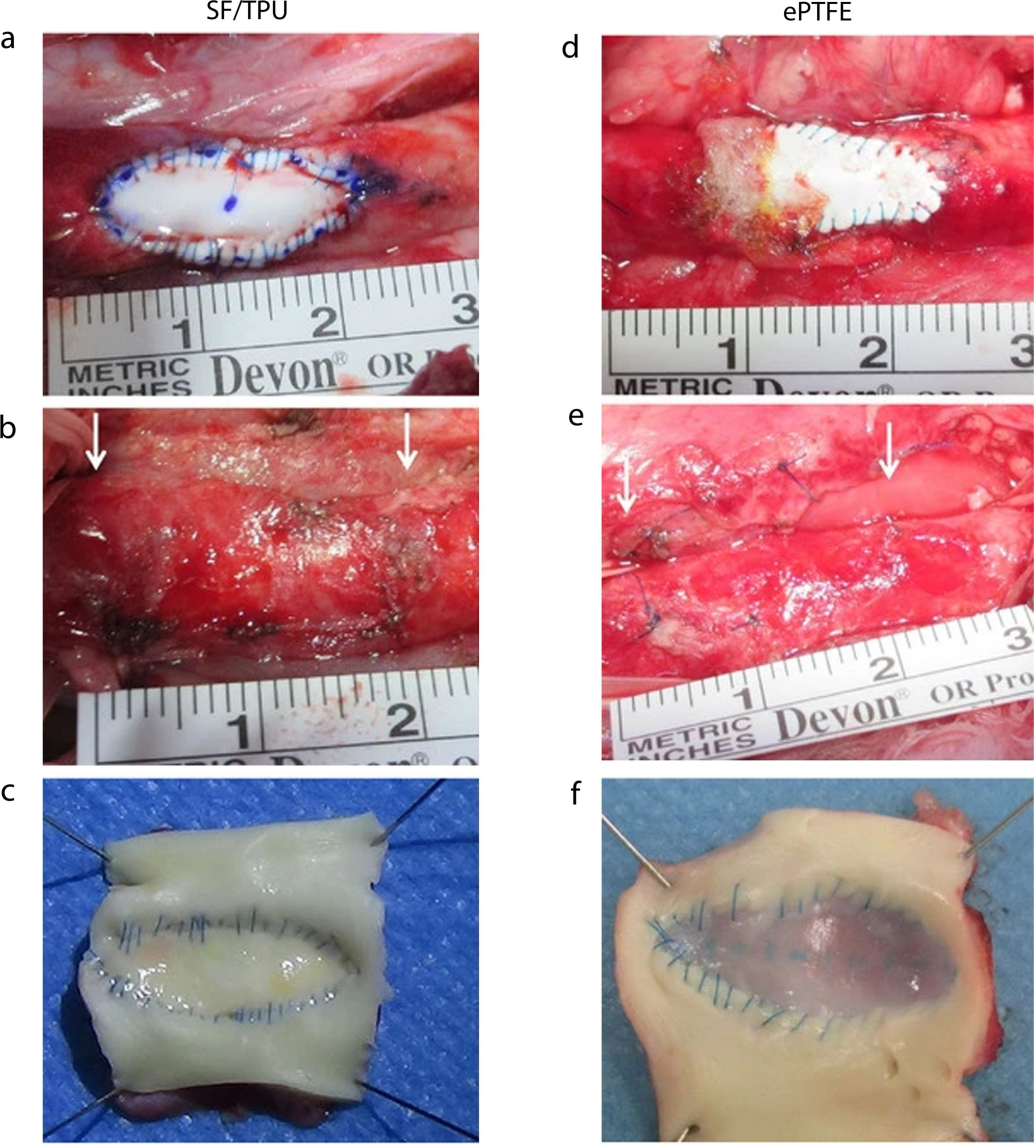
Implanted patches in a canine aorta. (a–c) SF/TPU and (d–f) ePTFE patches, where (a) and (d) show the patches at the time of implantation, while (b) and (e) depict the patches upon removal after 3 months. The white arrows indicate the proximal and distal edges of the implanted patch. (c, f) Luminal surface of the removed patches. Reprinted from [187], (R. Shimada et al., “Development of a new surgical sheet containing both silk fibroin and thermoplastic polyurethane for cardiovascular surgery“, Surgery Today, vol. 48, pages 486–494, published by Springer Nature, 2017, reproduced with permission from SNCSC). This content is not subject to CC BY 4.0.

A recent research work used TPU and SF to electrospin specialized biomaterials for vascular applications, as shown in Figure 6. In this study, Shimada et al*.* investigated the shortcomings of the surgical sheets used in congenital cardiovascular operations, including pseudointimal proliferation, calcification, and material deterioration. Using electrospinning, they developed a novel sheet of SF and TPU to create a non-woven nanofiber structure. The mechanical characteristics of the SF/TPU sheet, such as its flexibility, water permeability, and suture retention strength, were determined to be comparable or better to those of the current material, that is, expanded polytetrafluoroethylene (ePTFE). A canine descending aorta wall section was replaced with the SF/TPU sheet to verify biocompatibility. Three months later, a histological investigation was conducted. Excellent integration with the vascular wall, no bleeding from needle holes, well-restored intimal tissue on the patch’s surface, no calcium deposits, and minimal inflammation were observed. According to the results, the SF/TPU sheet has better mechanical qualities and tissue compatibility, which calls for longer-term research to validate its surgical application potential [187].

In another study, Shimada et al. formulated a patch material for aortic surgical patching by using a SF/PU blend in a 1:1 weight ratio to combine the biocompatibility of SF and the elasticity of PU. SF/PU patches were implanted into the abdominal aortas of rats, while ePTFE patches were kept as a control. Compared to ePTFE, the SF/PU patches were much easier to implant and more flexible. One week after implantation, the SF/PU patches already exhibited moderate levels of cell infiltration and collagen fiber accumulation, while the ePTFE patches exhibited none after three months. In addition, the ePTFE control group developed calcification after four weeks, which further confirms the most critical disadvantage of ePTFE patches. Throughout the study, the SF/PU patches did not show any significant signs of calcification [188]. Caldiroli et al. highlighted the incorporation of SF with PU to make a bio-hybrid material (Silkothane^®^), which seeks to address the challenges faced when applying autologous vessels in small-caliber vascular surgeries such as cardiac surgeries. They made vascular grafts that have an inner diameter of 1.5 mm using electrospinning. These grafts were evaluated against SF grafts as well as natural rodent blood vessels like the abdominal aorta and vena cava. These Silkothane^®^ grafts possessed a flexible randomized fibrous structure that closely resembled the size and structure of a rat’s aorta. Initial studies on Lewis rats showed that the grafts worked for small-caliber applications such as aortic insertion and femoral shunting. Three months after surgery, 94% of the grafts allowed for blood flow, and all rats survived the surgical procedure. Moreover, the study highlights the mechanical properties of blood vessels in rodents [189]. Kitpipatkun et al. focused on the biological complications and histopathological features by using electrospun SF/PU patches in diabetic (DM) and non-diabetic (control) rats. Diabetes was induced in the DM group with streptozotocin, and in both groups SF/PU patches were implanted in the abdominal aorta. The patches were assessed at post-implantation periods of one, two, three, and four weeks. The results indicated that the DM group exhibited more fibrosis formation and delayed endothelialization than the control group, but neither group showed signs of chronic inflammation. The fibrosis under hyperglycemic conditions was found to support the formation of new vascular structures, such as endothelial and vascular smooth muscle cells within the implanted patch [190].

### Future perspectives

The combination of electrospun PU and SF membranes is a game-changing approach in the field of wound healing and skin substitutes. Despite great progress in materials construction and design, there remains large potential for further research. Future investigation should be directed toward enhancing the biological activity of these membranes by using bioactive functional components, including antimicrobial nanoparticles, growth factors, or exosomes derived from stem cells, that would increase healing while decreasing the chances of infection. Another area that holds great promise is the design of membranes with multilayered or hybrid structures that meet specific wound requirements. These membranes may be capable of overcoming the problems associated with single-layer designs such as lack of mechanical strength, controlled biodegradation, and moisture balance for optimal ECM mimicry. Furthermore, novel electrospinning methods like 3D electrospinning or coaxial spinning can be applied to construct more advanced geometries of the fibers. These would provide higher strength and direct cell contact orientation, which are so important for nerve and blood vessel reconstruction.

Maintaining cost-effectiveness while increasing production is essential for commercial feasibility, particularly with the increased need for light-activated biomaterials. At the same time, looking for biocompatible, renewable precursors for the electrospinning process could solve some ecological issues while ensuring that the process remains environmentally sustainable. Comprehensive in vivo and clinical trials will be critical in bridging the gap from the laboratory to the field, ensuring that these membranes are effective as well as safe and comfortable for a broad range of clinical applications. The addition of intelligent materials such as stimuli-responsive PU/SF composites could enable even greater functionality, including controlled release, in vivo sensing, and other forms of active treatment. In addition, those working at the intersection of biomaterials science, medicine, and engineering will be able to maximize the functional capabilities of these membranes. Continuously evolving PU/SF electrospun membranes have the potential to revolutionize wound care through improved treatment efficiency and flexibility for enhanced recovery.

## Conclusion

Wound healing is a complex process that involves the interaction of many cells, ECM components, and bioactive chemicals to restore the damaged epidermis successfully. Finding a safe and convenient skin substitute made from synthetic or organic biomaterials capable of producing a dermoepidermal layer that can be implanted in a single procedure is of tremendous interest in biomedical science. For that matter, electrospun PU-based composites and their dressings have been applied in various forms, such as hydrogels, scaffolds, films, electrospun mats, and sponges to create a suitable microenvironment for all phases of wound healing. Combining SF and PU in electrospun membranes improves cell adhesion and proliferation, enhances cellular response, and provides excellent biocompatibility and mechanical strength. According to the existing literature, PU/SF-based composites are useful for encouraging tissue regeneration and as scaffolds to facilitate the formation of new tissue. Therefore, these composites could be promising materials for reconstructing soft tissues. Additionally, these blended fibers provide a low-cost and simple method to create innovative biomedical products. The combination of silk and polyurethane in electrospun membranes is a big step forward in the development of biomaterials, and it is anticipated that SF/PU-based composites will eventually be commercially important for wound healing and skin regeneration. To fully realize the potential of these composites in biomedical applications, future research should concentrate on refining the production processes and investigating the medical applications of these materials.

## Data Availability

Data sharing is not applicable as no new data was generated or analyzed in this study.
